# Experiencing Violence from Animal Owners in Veterinary Medicine: Results of a Nationwide Survey

**DOI:** 10.3390/healthcare14020262

**Published:** 2026-01-21

**Authors:** Irina Böckelmann, Beatrice Thielmann

**Affiliations:** Institute of Occupational Medicine, Faculty of Medicine, Otto von Guericke University Magdeburg, 39120 Magdeburg, Germany; irina.boeckelmann@med.ovgu.de

**Keywords:** vets, aggression, violence, conflict, stress

## Abstract

**Background/Objectives**: Veterinarians are among the most stressed of all professional groups. Their work is characterised by long working hours, high emotional demands and an increased risk of anxiety, depression, suicide and burnout. The aim of this cross-sectional study that examines retrospective records of experienced violence was to analyse the frequency of violent acts and their connection to certain factors (age, gender, place of work, and specialist area according to animal species). **Methods**: This nationwide, cross-sectional, online survey of veterinarians in Germany was conducted between July 2021 and February 2023. A total of 1053 veterinarians were included in the analysis, which was conducted according to the respondents’ age, (<35 years, 35–45 years and >45 years), gender, workplace and veterinary specialisation. Sociodemographic and work-related data were collected, as were responses to questions regarding experiences of violence, which were differentiated between verbal abuse and physical violence. The data were analysed using descriptive statistics and non-parametric group comparisons (Kruskal–Wallis test with Bonferroni correction, Mann–Whitney U test and Pearson’s chi-squared test). **Results**: Overall, 52.7% of veterinarians reported experiencing verbal abuse or physical violence at the hands of animal owners. Verbal abuse occurred, on average, more than three times per month, whereas physical violence was rare. Physical violence occurred significantly more frequently among middle-aged veterinarians (*p* < 0.001). The highest prevalence of verbal abuse or violence (72.5%, *p* < 0.001) was reported by veterinarians working in public authorities, while the lowest was reported by those working in laboratories. **Conclusions**: Workplace violence against veterinarians is a frequent occupational burden in Germany and highlights the urgent need for targeted prevention, de-escalation training and organisational support across veterinary settings.

## 1. Introduction

Numerous international studies confirm that veterinarians are exposed to various work-related stressors [1,2,3,4] and that these stressors are sometimes associated with long-term mental health problems, such as burnout, depression and suicidal tendencies [3,5,6,7,8,9,10,11]. Conflicts with animal owners and their high expectations, as well as the high level of responsibility for one’s own decisions in the treatment process, are often cited as reasons for mental stress in the workplace [12].

**Figure 1:**
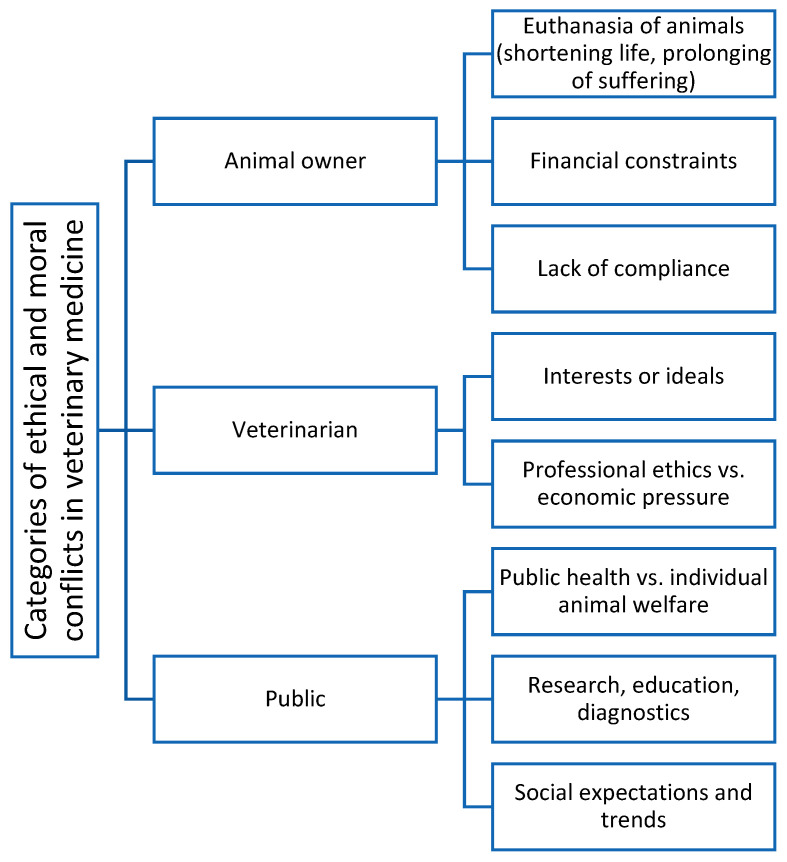
Exploratory conceptual framework of potential conflicts in veterinary medicine.

The exploratory conceptual framework shown in Figure 1 was further developed by the authors to illustrate and categorise potential sources of conflict in veterinary medicine. The framework is based on theoretical considerations and the authors’ professional experience in occupational medicine, and a synthesis of the relevant literature on ethical conflicts, occupational stress and interactions between veterinarians and clients. As various categories of ethical and moral conflicts in veterinary practice are currently identified [13], such conflicts can initially be classified according to their origin, which may be the animal owner, the veterinarian the public, or society.

The following potential conflicts originate with the animal owner [14,15,16,17,18]:

- *Euthanasia of animals:* This conflict arises when the animal owner wishes or even demands that one or more animals be killed, for example, to relieve pain and end the animal’s suffering, without there being any medical or objective reason for doing so (“shortening of life”) [14]. Another example of a conflict situation would be if the animal owner refuses to adhere to the necessary action for incomprehensible reasons (“prolongation of suffering”) and thus, despite medical indications, contradicts the conviction of the veterinarian, who is acting on the basis of evidence-based medical standards [15].
- *Financial constraints:* Such conflicts arise, for the most part, when a medical examination and/or treatment of an animal is necessary but the animal owner cannot or will not pay for it [16,17].
- *Lack of compliance:* This type of conflict arises when the veterinarian experiences a moral conflict due to a pet owner’s failure to comply with medical instructions that are therapeutic or preventive measures necessary to maintain the animal’s welfare, but the pet owner behaves contrary to the moral beliefs and ethical ideals of the veterinarian [18].

On the part of veterinarians, the following potential conflicts arise [17,19,20,21]:

- *Interests or ideals:* This includes several possible situations. For example, the veterinarian’s personal interests, which are actually secondary, may outweigh their professional or social duties and guidelines with respect to veterinary medicine [19] or their own moral ideals and convictions (i.e., they must choose between the two). Another example of this would be if external authorities require the veterinarian to perform a medical procedure that contradicts his moral beliefs about treating animals with respect and care or prevents him from enforcing his personal moral convictions [20].
- *Professional ethics* vs. *economic pressure:* Veterinarians are caught between professional ethics and economic pressure [17,21]. On the one hand, their profession obliges them to put animal welfare above all else, but on the other hand, they have to run their practices as businesses. This conflict is evident, for example, in cost-intensive emergencies, such as colic surgery on a horse, which costs several thousand euros and for which the bill is not paid afterwards. In such cases, the practice is left to bear the costs, even though the veterinarian’s actions are medically appropriate and in the best interest of the animal. This often leads to moral stress when economic losses collide with ethically necessary decisions. Thus, the conflict between responsibility towards the animal and financial reality becomes a central problem in everyday veterinary practice.

Finally, the potential for conflict arising from public opinion and society’s expectations must be considered [20,22,23,24,25,26,27]:

- *Public health* vs. *individual welfare of the animal:* Another area of conflict arises from the tension between public health and the individual welfare of the animal. Veterinarians have a dual responsibility: they must protect the individual animal and simultaneously contribute to the preservation of public health. For example, when dealing with zoonoses or notifiable animal diseases (for example, avian influenza), it may be necessary to isolate or kill animals to prevent further spread [20,22]. Such measures often conflict with the veterinary ethos of preserving life and avoiding suffering. Moreover, there is a clear expectation in society that public health should be comprehensively protected. This obligation is also enshrined in law in the Animal Welfare Act and its associated regulations.
- *Research, education and diagnostics:* Another area of conflict exists in the field of research, education and diagnostics. Here, animals are used for scientific, educational or diagnostic purposes, which are often associated with stress or restrictions on the well-being of the animals [23]. Even if such interventions serve medical progress or the protection of the general public, they conflict with the principle of avoiding suffering of animals. The Animal Welfare Act strictly regulates the conditions under which animal experiments may be approved and requires the testing of alternative and complementary methods [24]. Nevertheless, moral conflict remains, as the benefits to science and society must always be weighed against the individual suffering of the animals concerned.
- *Social expectations and trends:* Another area of conflict arises from the increasing social expectations placed on veterinarians. Today, they are expected not only to have professional competence but also to take social responsibility, for example, in matters of sustainability, animal welfare, nutrition and the responsible use of antibiotics [25,26,27]. Veterinarians are expected to educate the public, identify abuses and contribute to ethically acceptable animal husbandry. However, this role may conflict with the individual ideas or economic interests of animal owners, for example, when certain husbandry conditions or treatments are critically evaluated from a professional perspective [25]. Social trends, such as increasing sensitivity to animal welfare or plant-based nutrition, also influence expectations of veterinary practices. In recent years, there has been a marked increase in public concern about animal welfare, as reflected in consumer attitudes and policy debates. This concern has been shown to influence expectations regarding the treatment of animals in various contexts, ranging from companion animals to livestock production systems [26]. At the same time, the growing popularity of vegetarian and vegan diets—which, according to population surveys, are associated with stronger pro-animal welfare attitudes—reflects broader societal shifts in how humans view and relate to animals. This influences the demands placed on veterinary care and the ethical conduct of practitioners [27].

These diverse areas of conflict show that veterinary practice is often accompanied by moral and emotional stress and make it clear that veterinarians have to make difficult decisions every day between ethics, empathy and economic reality. These decisions can be, in some cases, quite challenging for them personally.

The development of ethical guidelines in veterinary medicine and the integration of ethical aspects into the training of veterinarians help guide veterinary decision-making during dilemmas and ensure that the welfare of the animal is always paramount. The decision of whether and, if so, when to euthanise an animal is extremely difficult and requires knowledge and experience with fundamental ethical categories and values.

**Figure 2:**
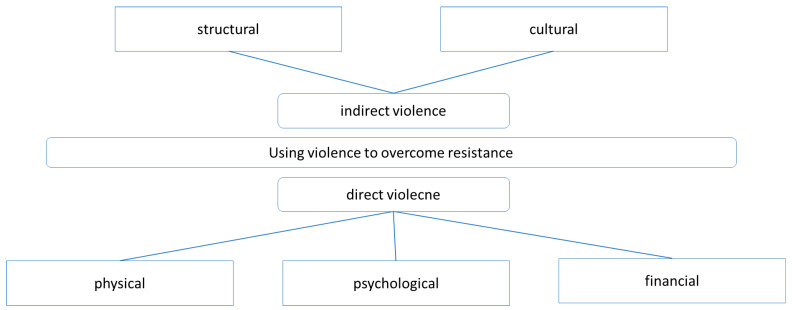
Forms of violence according to Grond 2007.

Conflicts with animal owners can ultimately escalate into violent situations. Grond [28] distinguishes between direct and indirect violence (Figure 2).

As part of a study on stress and strain among veterinarians in Germany [29] we analysed the relationships between working conditions, stress and health consequences with respect to this occupational group. This yielded initial findings on the topic of “experienced violence” in the field of veterinary medicine [12], with 52.8% of the 1035 veterinarians surveyed indicating that they had experienced violence or verbal abuse from animal owners during their daily working lives. When asked, “When you experience violence or verbal abuse from pet owners, how much does it affect you”, 6.8% responded, “Not at all” (0.5%) or “Hardly at all” (6.3%); 20.1% responded, “Moderately”; 34.8% responded, “Significantly”; and 38.4% responded, “Very significantly”. When asked about the number of violent incidents experienced in the workplace, which is also described in the literature as extremely stressful for veterinarians, 25% of the respondents reported that they have experienced more than three cases of verbal abuse per month, while 11.4% responded that violent incidents occurred more than once a month in their practice. On average, the number of verbal abuse incidents experienced in the workplace was 3.2 ± 4.54 per month (median 2; min–max 0–50), and the number of violent incidents experienced was 0.2 ± 0.73 (median 0, minimum 0, maximum 10) cases per month.

A literature review revealed that there is insufficient data on violence, physical and verbal assault, and aggression in the professional environment of German veterinarians, and only a few findings are available from other countries [30]. In addition to the need for further research, there is a need to develop recommendations for action for veterinarians.

Based on the initial findings on the topic of “experienced violence”, the following questions were formulated: Is the frequency of violent acts associated with certain factors (age, gender, place of work, specialist area according to animal species, etc.)? Are there differences in the perception of the degree of stress caused by violent acts in conflict situations? This study addresses only the category of direct violence, specifically, physical and psychological (verbal) violence.

## 2. Materials and Methods

The entire research project “Causes and consequences of psychological stress in everyday work and in emergency services regarding the veterinary profession in the Federal Republic of Germany” was submitted in advance to the Ethics Committee of Otto von Guericke University Magdeburg. The positive vote is available under number 91/21. The study was registered with the German Register of Clinical Studies under number DRKS00026106 and funded by the Professional Association for Health and Welfare Services (grant no. 1544). The content and details of the study are presented in detail in the published study protocol [29].

### 2.1. Recruitment and Subjects

Study participants were recruited via the study information flyer, which was available on the websites of the German Veterinary Medical Association and the state veterinary medical associations. In addition, targeted requests for assistance in recruiting potential participants were sent to veterinary professional associations, societies and other relevant institutions. Further information was also published in the journal “Deutsches Tierärzteblatt” (issue 09/2021) and via social media channels. For this reason, a response rate cannot be determined.

### 2.2. Subjects

A total of 1053 veterinarians aged between 23 and 79 voluntarily participated in the online survey regarding the occurrence of violence. The sample consisted of 373 men (35.4%) and 680 women (64.6%) from different specialist areas according to animal species (small animals (55%), large animals/farm animals and horses (17.8%), small animals and large animals (14.8%), laboratory work (3.2%) and government agencies (9.0%)). The veterinarians were employed in various types of positions, i.e., self-employed (40.5%), public sector employees (9.8%), practices/clinics (15.9%), civil servants (3%), assistant doctors (25.5%), doctoral students (0.8%), private sector (2.8%), etc. The place of work in 16 federal states varied greatly, specifically, large cities (26.9%), medium-sized and small towns (33.4%) and rural areas (39.7%).

The inclusion criteria for participation in the survey included the participant’s voluntary participation in the study and professional activity as a veterinary staff member. Veterinarians with less than one year of professional experience were excluded from the study.

### 2.3. Survey

A quantitative survey to analyse the relationship between working conditions, stress, and health consequences among veterinarians was conducted online between July 2021 and February 2023. The web application SoSci Survey version 3.2.03-i (SoSci Survey GmbH, Münich, Germany) was used to create the online questionnaires.

As part of the abovementioned questionnaire, socio-demographic and work-related data were gathered and questions were asked about the respondents’ experiences of direct violence (in the form of physical violence or verbal abuse/psychological violence) by pet owners, i.e., whether it occurred, how severe it was (from 1 “Not at all” to 5 “Very severe”) and how often it occurred per month.

### 2.4. Statistics

The data from the SoSci Survey were evaluated descriptively and in mean group comparisons using the Statistical Package for the Social Sciences (SPSS, Version 28, IBM, Armonk, NY, USA).

Descriptive statistics were used to calculate frequencies and percentages for categorical variables, as well as means, standard deviations, medians and ranges for continuous variables.

Group-level comparisons were performed to examine differences in the prevalence and frequency of verbal abuse and physical violence, as well as the perceived level of stress, according to age group, gender, place of work and veterinary specialisation. Non-parametric tests were applied due to non-normal data distributions. Differences between multiple groups were analysed using the Kruskal–Wallis test with Bonferroni correction, and Mann–Whitney U tests were used for comparisons between two groups. Associations between categorical variables were examined using the Pearson chi-squared test. A *p*-value of less than 0.05 was considered statistically significant.

## 3. Results

The 1053 veterinarians, on average, 41.8 ± 10.17 years of age, with 33% (*n* = 348) under 35 years of age (AG I), 34% (*n* = 361) between 36 and 45 years of age (AG II), and 33% (*n* = 344) were 46 years of age or older and belonged to AG III.

The group of younger veterinarians had been working for 4.7 ± 2.78 years (median 5), the middle-aged veterinarians reported 12.7 ± 4.81 years (median 13) and the older veterinarians had 25.5 ± 6.92 years (median 25) of professional experience (p_Kruskal-Wallis_ < 0.001).

The gender distribution in the three age groups was significantly different (p_χ_^2^_according to Pearson_ < 0.001), with more women among younger veterinarians (70.7%) than among middle-aged veterinarians (66.2%). The smallest proportion of female veterinarians, 56.7%, was found in AG III.

To answer the question of whether incidents of violence are more frequent in certain age groups or among certain genders, the results were analysed for each of the three age groups and differentiated by gender (Table 1).

When considering all incidents of violence, 56.7% occurred among those in AG I; 52.8% among those in AG II; and 48.7% among those in AG III. Although younger veterinarians seemingly experienced more verbal abuse or violence, the differences were not significant (p_χ_^2^_according to Pearson_ = 0.109). No significant differences were found between the gender groups (p_χ_^2^_according to Pearson_) = 0.930).

**Figure 3:**
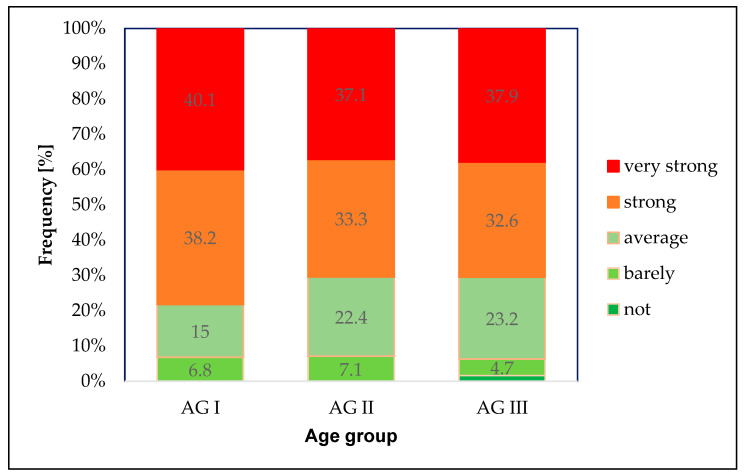
Degree of stress: relative frequency (%) of responses regarding the degree of stress caused by the perceived experience of verbal abuse or violence (“… stresses me…”) in three age groups (AG I, AG II and AG III).

The distribution of the degrees of stress perceived as a result of experiencing verbal abuse or violence was also not significantly different across age groups (p_χ_^2^_according to Pearson_ = 0.109) and was comparable to the distribution in the overall sample (Figure 3). In AG I, 78.3% of the respondents estimated that they were severely or very severely stressed by these events, whereas the figures were slightly lower in AG II (70.4%) and AG III (70.5%) groups.

**Figure 4:**
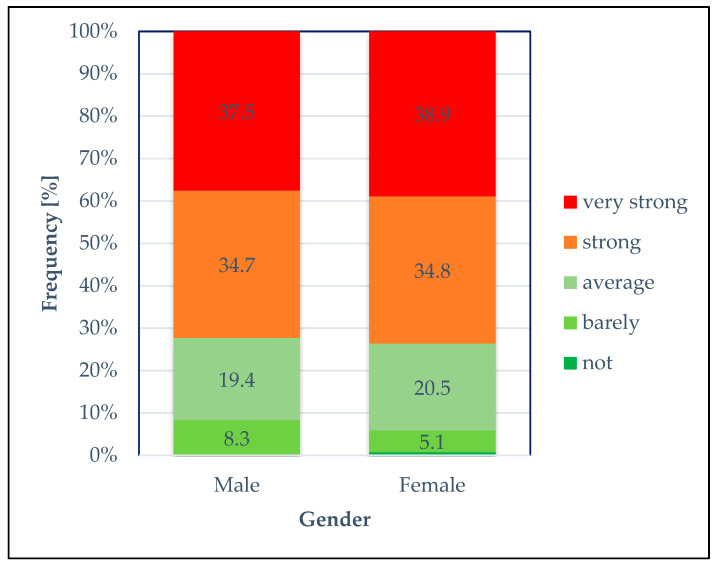
Degree of stress: Relative frequency (%) of responses regarding the degree of stress caused by the perceived experience of verbal abuse or violence (“… stresses me out…”) in gender groups.

Similar results were found in the comparison between the gender groups (Figure 4). Male and female veterinarians felt similar levels of stress (p_χ_^2^_according to Pearson_ = 0.393), with 63.7% of the female veterinarians surveyed rating their experience of violence as “strong” or “very strong”, while 72.2% of the male respondents rated their experience as “strong” or “very strong”.

The number of verbal abuse incidents (verbal violence) in everyday working life (Table 2) was statistically comparable across the three age groups (p_Kruskal-Wallis_ = 0.921) and ranged between 3.0 ± 3.79 cases per month (AG I) and 3.7 ± 5.88 cases per month (AG II). Physical violence occurred significantly more frequently (p_Kruskal-Wallis_ < 0.001) among veterinarians in AG II (0.3 ± 0.87 cases per month) than in AG I (0.1 ± 0.22; p_Bonferroni_ < 0.001) and AG III (p_Bonferroni_ = 0.015).

The gender group comparison (Table 2) revealed no significant differences (p_Mann-Whitney_ = 0.875 verbal abuse and p_Mann-Whitney_ = 0.155 physical violence).

A closer look at the data on experiences of verbal abuse or violence by animal owners in the various specialist areas by animal species (Table 3) revealed that almost 75% of veterinarians working in public authorities reported having experienced violence or verbal abuse (72.5%). The lowest proportion of “yes” responses to experiencing verbal abuse or violence in their everyday working lives was among veterinarians working in laboratories (10%), followed by veterinarians who treat large animals (farm animals and horses) (26.1%). More than half (63.0%) of veterinarians who treat small animals confirmed that they had experienced violence or verbal abuse in their everyday working lives. These differences between the specialist areas according to animal species were significant (p_χ_^2^_according to Pearson_ < 0.001).

**Figure 5:**
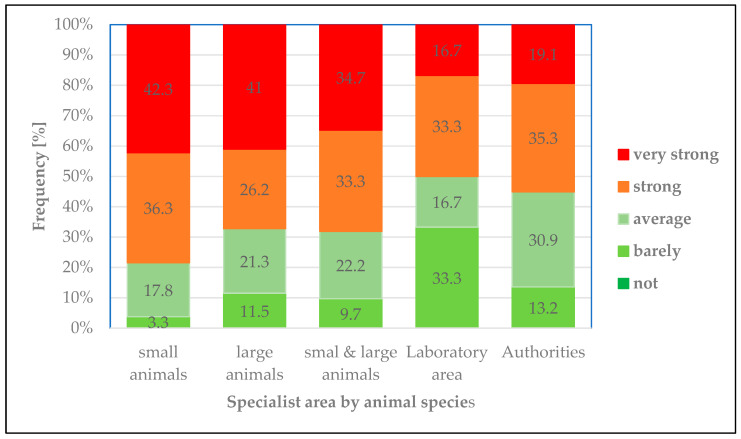
Degree of stress: Relative frequency (%) of responses regarding the degree of stress caused by perceived experiences of verbal abuse or violence (“… stresses me out…”) in different specialist areas by animal species.

The incidents of physical and verbal violence were perceived to varying degrees by veterinarians from different fields (p_χ_^2^_according to Pearson_ < 0.001). Veterinarians working in government agencies, who were confronted with conflicts more often, reported being less affected by the altercations (Figure 5). Conversely, 54.4% of veterinarians in public authorities rated their experiences of violence as “high” or “very high”. However, the highest proportion of veterinarians (78.6%) with the highest levels of stress was found among veterinarians in small animal practices.

The differences in the number of verbal abuse incidents experienced in everyday working life (Table 4) were statistically significant between veterinarians in different specialties (p_Kruskal-Wallis_ < 0.001). For example, with respect to veterinarians working in government agencies, incidents occurred on an average of 3.8 ± 4.22 times per month, whereas they occurred only 0.9 ± 1.46 times per month in laboratories. These differences between the two groups are statistically significant (p_Bonferroni_ = 0.012). Further statistically significant differences were found between the authority group and large animal group (p_Bonferroni_ < 0.001), between the small animal and laboratory (p_Bonferroni_ = 0.021), and between the small animal and large animal group (p_Bonferroni_ < 0.001). The number of incidents of physical violence experienced also differed significantly among veterinarians in the five specialist areas (p_Kruskal-Wallis_ = 0.034).

When analysing the data on experiences of verbal abuse and violence by animal owners based on the place of work, statistically significant differences were found (p_χ_^2^_according to Pearson_ < 0.001). Similarly, significant differences were found among the “yes/no” responses (Table 5). For example, 62.3% of veterinarians from large cities stated that they had experienced violence or verbal abuse at least once while in rural areas, the percentage of “yes” responses was significantly lower at 43.5%.

**Figure 6:**
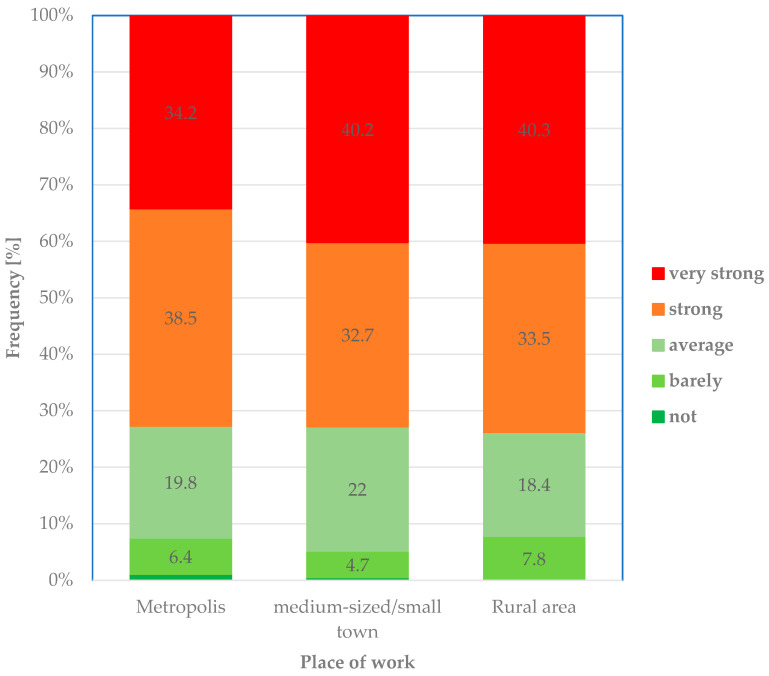
Degree of stress: Relative frequency (%) of responses regarding the degree of stress caused by the perceived experience of verbal abuse or violence (“… stresses me out…”) among veterinarians in different workplaces.

The cases of verbal abuse or violence experienced by veterinarians at different workplaces were statistically comparable (p_χ_^2^_according to Pearson_ = 0.550) and were perceived as “severe” or “very severe” (Figure 6). Approximately 73% of veterinarians in the respective workplace groups stated that they were severely to very severely affected by experience.

The number of cases of violence experienced (verbal or physical) were also statistically comparable across groups of veterinarians in different work locations, i.e., p_Kruskal-Wallis_ = 0.065 for verbal abuse and p_Kruskal-Wallis_ = 0.255 for physical violence. See Table 6.

Given that fewer than ten veterinarians participated in Berlin, Bremen, Mecklenburg–Western Pomerania and Saarland, no statistics were compiled for comparison between federal states.

## 4. Discussion

This study examined the frequency and characteristics of verbal and physical violence experienced by veterinarians, analysing differences according to age, gender, place of work and veterinary specialisation. Overall, more than half of the surveyed veterinarians reported experiencing workplace violence or verbal abuse from animal owners, with verbal abuse occurring considerably more frequently than physical violence. While the prevalence of violence and the perceived stress associated with it were comparable across age and gender groups, middle-aged veterinarians reported significantly higher frequencies of physical violence. Significant differences were observed between veterinary specialisms, with the highest prevalence reported among those working in public authorities and the lowest among laboratory veterinarians. Despite experiencing violence more frequently, veterinarians working in public authorities perceived these incidents as less stressful than those working in small animal practices.

Studies indicate that violence in the workplace is more common in the health and social services sector than it is in other industries [31], and that such aggression and violence in the workplace affect the well-being of employees [32,33], which ultimately results in a lower quality of life and can contribute to job dissatisfaction [34] and early career abandonment [28,35,36]. Accordingly, research is needed to analyse the psychological consequences of workplace violence. The “pet boom”, especially during the pandemic [37], exacerbated the general professional stress situation in veterinary medicine, while at the same time, there was a shortage of doctors and a decline in practices [38].

In addition to the well-known categories of ethical and moral conflicts in veterinary practice, dissatisfaction with veterinary care can be a further source of conflict [13].

The results of this study reveal that veterinarians experience an average of more than three incidents of verbal abuse or violence in the workplace per month, although physical violence is less common than verbal forms of violence.

Acts of verbal and physical violence were experienced with comparable frequency by veterinarians across all three age groups and by both women and men in their everyday working lives, and the events were perceived as comparably stressful. Only the number of acts of physical violence was statistically significantly greater among veterinarians in AG II than among their younger and older colleagues. The quantitative data on the number of cases experienced per month were similar for both gender groups.

Although veterinarians working in veterinary authorities experienced such acts of violence more often in their everyday work, the events did not affect them to the same degree as such events affected, for example, veterinarians working in small animal practices. The reason for the frequent reports of acts of violence among the authorities may be due to the type of the animal owner. In most cases, these are farmers who keep animals, and the veterinary authorities often make decisions that are of existential importance for animal owners. If abuses are discovered in the stables of a farm that keeps animals, if mistreatment of large animals is reported or if a lack of veterinary care is proven, the veterinary authority must take legal action, which may include orders, inspections, decrees, herd reductions or bans on animal husbandry. These decisions by official veterinarians can trigger an escalation of anger or frustration on the part of the animal owner that can then lead to conflicts. The same applies to conflict situations between veterinarians in veterinary authorities and food business operators. Veterinarians employed in food chain businesses are responsible for both animal welfare and food safety. The recommendations for implementing the “Code of Ethics for Veterinarians in Germany” [19] highlight the challenges and tasks of veterinarians who are responsible for animal welfare and the highest level of food safety in the food chain. In food production, in particular, there may be conflicts of interest between food producers and consumers on the one hand and animal welfare requirements on the other as food safety must be guaranteed while ensuring the highest possible level of animal welfare. If deficiencies in animal husbandry, animal care or food production are identified, veterinarians must point this out to the animal owner and support them in remedying these deficiencies. Conflict situations can arise if the animal owner is not reasonable and/or unwilling to remedy animal welfare issues and if the veterinarian involves the relevant government authorities. However, veterinarians may also refuse to treat animals because of inadequate husbandry conditions, which can lead to conflict. In these cases, veterinarians provide intensive support and advice to animal owners on possible measures to promote biosecurity to protect livestock, humans and the environment. Conflict potential is also conceivable in the breeding of animals that provide food. Among other things, this raises the question of whether animals may not be used for economic reasons on the basis of their sex. Veterinarians support breeding goals and uses that ensure the physical integrity of the animals and allow both sexes to be used. Potential conflicts can also arise during slaughter and death. Veterinarians ensure that all persons involved in the drive and stunning of animals treat them calmly and gently before killing them to avoid causing them fear. If there is a situation that violates animal welfare (e.g., during transport to the slaughterhouse, during stabling at the slaughterhouse, in the mistreatment of slaughter animals or in the improper stunning and bleeding of these animals) veterinarians must take immediate action to remedy the abuses that have been uncovered.

Veterinarians in large cities report frequent acts of violence and verbal abuse, and these experiences affect them just as much as the experiences affect their colleagues in medium-sized and small towns or rural areas. Whether this frequent occurrence is due to the so-called “anonymity of pet owners” in large cities remains a matter of speculation. In rural areas, access to the nearest veterinary practice may be limited or difficult, primarily due to longer travel distances and the smaller number of veterinarians practising in these regions.

### 4.1. Recommendations for Action

The results emphasise the need for occupational health and safety measures that specifically address stress factors. In addition to the necessary risk assessment (including psychological stress), measures should be taken to reduce stressful situations and strengthen veterinarians’ resources. Violence prevention refers to a targeted and successful approach to reducing and, if possible, preventing violence [39]. In this context, interventions that strengthen resilience, promote coping strategies and/or support the learning of defence mechanisms can be helpful. This necessity should be explained in further education/training events. Possible topics include recognising and correctly assessing dangers and sources of risk, violence prevention, professional communication, de-escalation strategies in crises, knowledge of self-protection in crisis situations, professional defence strategies against attacks (including setting boundaries), basic knowledge of the law (powers, rights and obligations, especially of veterinarians in veterinary authorities) and protective measures, as well as ethical reflection. Ethical skills should be taught in a targeted and practical manner. Targeted training measures and specific qualifications in the field of violence prevention and de-escalation can prevent aggressive situations in veterinary medicine from leading to excessive stress and overload for veterinarians. Accordingly, veterinary staff should be trained to address violence professionally and initiate preventive and de-escalating measures. It is recommended that such training measures be taught during veterinary studies as this would be the optimal time to teach resilience training and skills and to educate students about the concepts and specific techniques of well-being as aspects of professionalism. All of these factors are crucial for professional efficiency and stamina. It is also important to learn during one’s studies how to strengthen personal resources, how to follow up and debrief on incidents of aggression and violence in a professional setting, and how to tailor care to the needs of those affected. Hence, the following points are important: rapid processing of the trauma, offers of conversation (e.g., by peers, colleagues or friends), sufficient time for the recovery process, no stigmatisation, and ensuring the physical safety and psychological well-being of those impacted by the event [40,41,42]. Some studies have found that there are online interventions for violence prevention that were able to raise awareness of violence among nursing students, and participants stated that they found the content useful strongly recommended that the course be included in nursing curricula [43].

It is, therefore, particularly important that veterinary staff receive adequate training in theoretical knowledge and practical skills in the areas of prevention, crisis management and follow-up care for aggression and violence. It is equally important to recognise one’s own reactions to aggression and violence to be able to respond appropriately. Theoretical background knowledge regarding aggression and violence is helpful and important in this regard [44].

Primary prevention involves approaches designed to prevent violence from occurring in the first place. This means identifying risks at an early stage and creating conditions that promote nonviolence. These approaches do not differ significantly from those used by nursing professionals and doctors in emergency departments [45]. These include access controls, secure registration areas, a safe place of refuge, an emergency plan, an aftercare concept for cases of experienced violence, and the creation of peer support [45]. Learning de-escalating communication styles and developing empathy are also part of this process.

Secondary prevention involves approaches that involve the immediate response to violence (i.e., in an emergency, de-escalating intervention is needed) to end aggression and violence [46].

The focus of tertiary prevention is on long-term care after violent incidents with the aim being to reduce or eliminate trauma associated with violence and prevent relapses and posttraumatic stress disorder through follow-up care. By analysing the violent incidents, strategies can be developed to prevent similar situations in the future. Furthermore, discussing and processing violent incidents within the team helps reduce the stress caused by violent behaviour on the part of animal owners and enables a joint learning process to develop a professional approach to dealing with aggressive situations [47].

In the healthcare sector, international professional associations have formulated guidelines for protection against harassment and violence because of the relevance of aggression and violence in the workplace [48]. This has led to a reassessment of the culture of error. In this context, a culture of error means that incidents of violence must be communicated with the aim to find solutions, not to find those responsible [49].

This study finds that violence (whether verbal or physical) is not an acceptable occupational hazard in the veterinary profession, that, accordingly, a zero-tolerance policy towards violence and threats is essential, and that employers are legally obliged to provide comprehensive protection for their employees in accordance with their duty of care. However, depending on the field (small animals, large animals/farm animals and horses, small animals and large animals, laboratories and authorities), these protective measures are implemented in very different ways. Additionally, clear legal frameworks are needed to ensure a safe working environment, especially in large, medium-sized and small towns. The recommendations of the veterinary association, such as the recommendation to implement the “Code of Ethics for Veterinarians in Germany”, form the basis for action for employees. Veterinarians should receive, on a regular basis, awareness-raising and training measures for dealing with and de-escalating conflicts and aggressive behaviours. Ideally, professional associations and accident insurance funds, such as the providers of German statutory accident insurance (DGUV), should support such measures. While currently, online courses are available from the BGW [50], face-to-face training courses should also be provided.

As the study by Böckelmann et al. (2024) revealed, many veterinarians enter the profession due to their idealistic love for animals [12]. Specifically, when asked about their reasons for choosing to study veterinary medicine, love for animals was by far the most popular answer, with 56.1% of the respondents citing this reason. However, the everyday working life of a veterinarian often looks quite different from the romanticised image of this profession. As the study shows, the downsides of being a veterinarian include verbal abuse and physical attacks. The media has also reported cases of negative reviews on internet portals as well as threats. Accordingly, this study calls for the creation of a register for emergency and reporting procedures related to veterinary medicine where violent situations can be reported. Moreover, these violent situations should be clearly defined so that veterinarians can correctly distinguish between different forms of violence, and the Veterinary Association and larger institutions should designate contact persons to whom veterinarians can turn in confidence.

Finally, it is also recommended that veterinarians and veterinary assistants be included in the protection set out in Sections 113–115 of the German Criminal Code (StGB). Veterinary professionals regularly face an increased risk of physical and psychological violence, particularly when providing emergency services or carrying out compulsory measures or official veterinary inspections. In these situations, they may be required to enforce legal regulations or make decisions that conflict with the interests of animal owners.

In such situations, veterinarians often find themselves in a position similar to that of other public service professionals, performing legally mandated tasks aimed at safeguarding animal welfare, public health, and food safety. Such activities often involve exercising authority or implementing statutory obligations, which can provoke aggressive reactions from those affected. As many veterinary activities can be classified as ‘activities serving the common good’, it is reasonable that veterinary staff should be granted special legal protection similar to that provided for other professions performing public duties. The application of enhanced protection in accordance with Section 46 of the German Criminal Code would recognise the societal importance of veterinary work and improve protection against violence in the workplace.

### 4.2. Limitations

One of the limitations of this study is its design (i.e., a cross-sectional study with a retrospective collection of incidents of violence). In other words, as it provides only a snapshot, no causal statements can be concluded. In addition, no further distinction is made among veterinarians working in public authorities as to whether they supervise animal transport; care for animals in sports, competitions and events; treat animals in wildlife parks, zoos and circuses; are involved in animal experiments; or care for animals in education, health, social services and law enforcement (police). Another limitation of the study is that participants were not given a standardised definition of verbal abuse. As verbal abuse can be more subtle and open to individual interpretation than physical violence, this may have resulted in variability in how participants identified and reported such experiences.

The subjective assessment of violent incidents by the respondents is a limitation of this study, as the perceived severity and significance of such events may change over time. Retrospective evaluations can be influenced by recall bias, whereby incidents may be perceived as more severe as a result of later reflection or cumulative stress, or conversely, as less significant due to coping strategies or habituation [51,52]. Furthermore, the period of time referred to in the question regarding the number of violent incidents per month was not specified in this study.

## 5. Conclusions

The results of this study show that violence and verbal abuse in the workplace are common occurrences among veterinarians in Germany. More than half of those surveyed reported having experienced such incidents. Verbal abuse is a regular occurrence in everyday working life, while physical violence is less common but more frequently reported in certain groups and areas of work. The significant differences between veterinary specialties and workplaces, as well as the high level of stress associated with these experiences, highlight the importance of addressing violence as a significant workplace health issue.

Primary, secondary and tertiary prevention measures, as well as crisis management and aftercare in cases of aggression and violence, that take into account the specific characteristics of the frequency of occurrence at certain workplaces and in certain specialist areas of veterinary medicine should be developed. There is still a need for further research with respect to violent behaviours of animal owners, strategies for dealing with conflict between veterinarian staff and animal owners, and ways to improve communication skills of veterinary staff when dealing with animal owners. In addition, a centralised recording of all incidents of violence is necessary to highlight the need for action in terms of organisational and political decisions.

In view of the increasing stress and incidents of violence against veterinary staff, effective measures for protection and prevention are urgently needed. Only through joint action can safe working conditions and respectful treatment be guaranteed.

As a society and as animal owners, we should be aware of this responsibility and treat veterinary work and the workers with more understanding, respect and trust.

## Figures and Tables

**Table 1:** Data on verbal abuse or violence experienced in the three age groups and in both gender groups.

Experiences of Verbal Abuse or Violence	AG			p_χ_^2^	Gender		p_χ_^2^
	I (*n* = 342)	II (*n* = 356)	III (*n* = 337)		Male *n* = 357	Female *n* = 678	
	Frequency (Number (%))				Frequency (Number (%))		
Yes	194 (56.7%)	188 (52.8%)	164 (48.7%)	0.109	189 (52.9%)	357 (52.7%)	0.930
No	148 (43.3%)	168 (47.2%)	173 (51.3%)		168 (47.1%)	321 (47.3%)	

Notes: AG = Age groups; AG I = Age under 35 years; AG II = Age 36–45 years; AG III = Age 46 years and older; _χ_^2^ = Chi-squared test according to Pearson.

**Table 2:** Experiences of verbal abuse or violence [number/month] in the three age groups and in both gender groups.

Verbal Abuse or Violence Experienced	AG			p_KW_ (p_B_)	Gender		p_MW_
	I (*n* = 180)	II (*n* = 162)	III (*n* = 116)		Male *n* = 177	Female *n* = 286	
	Mean ± Standard Deviation Median (Min–Max)				Mean ± Standard Deviation Median (Min–Max)		
Experience of verbal abuse [number/month]	3.0 ± 3.79 2.0 (0–30)	3.7 ± 5.88 2 (0–50)	3.3 ± 4.46 2 (0–30)	0.921 (-)	3.3 ± 4.07 2 (0–30)	3.3 ± 5.2 2 (0–50)	0.875
Experience of violence [mumber/month]	0.1 ± 0.22 0 (0–1)	0.3 ± 0.87 0 (0–9)	0.1 ± 0.36 0 (0–2)	<0.001 (I–II < 0.001 II–III 0.015)	0.17 ± 0.49 0 (0–4)	0.13 ± 0.63 0 (0–9)	0.155

Notes: AG = Age groups, AG I = Age under 35 years; AG II = Age 36–45 years; AG III = Age 46 years and older; KW = Kruskal–Wallis test, B = Bonferroni correction; MW = Mann–Whitney test.

**Table 3:** Data on verbal abuse or violence experienced in the specialist areas according to animal species.

Experiences of Verbal Abuse or Violence	Specialist Areas by Animal Species					p_χ_^2^
	Small Animals *n* = 576	Large Animals *n* = 184	Mixed Animals *n* = 154	Laboratory Area *n* = 30	Authorities *n* = 91	
	Frequency (Number (%))					
Yes	363 (63.0%)	48 (26.1%)	66 (42.9%)	3 (10%)	66 (72.5%)	<0.001
No	213 (37.0%)	136 (73.9%)	88 (57.1%)	27 (90%)	25 (27.5%)	

Notes: Mixed animals large and small animals; _χ_^2^ = Chi-squared test according to Pearson.

**Table 4:** Verbal abuse or violence experienced [number/month] in the specialities by animal species.

Experiences of Verbal Abuse or Violence	Specialist Areas by Animal Species					p_KW_	p_B_
	Small Animals *n* = 576	Large Animals *n* = 184	Mixed Animals *n* = 154	Laboratory Area *n* = 30	Authorities *n* = 91		
	Mean ± Standard Derivation Median (Min–Max)						
Verbal abuse [number/month]	3.7 ± 4.83 2 (0–50)	2.1 ± 4.29 1 (0–30)	2.9 ± 5.40 2 (0–40)	0.9 ± 1.46 0 (0–4)	3.8 ± 4.22 2 (0–20)	<0.001	Authority—Laboratory (0.012) Authority—large animals (<0.001) Small animals—Laboratory (0.021) Small animals—large animals (<0.001)
Violence [number/month]	0.2 ± 0.63 0 (0–9)	0.0 ± 0.18 0 (0–1)	0.1 ± 0.25 0 (0–1)	0.0 ± 0.00 0 (0–0)	0.3 ± 0.86 0 (0–4)	0.034	-

Notes: Mixed animals large and small animals; KW = Kruskal–Wallis test, B = Bonferroni correction.

**Table 5:** Information on verbal abuse or violence experienced by veterinarians in different work locations.

Experiences of Verbal Abuse or Violence	Place of Work			p_χ_^2^
	Large City (*n* = 273)	Medium/Small Town (*n* = 348)	Rural Areal (*n* = 414)	
	Frequency (Number (%))			
Yes	170 (62.3%)	196 (56.3%)	180 (43.5%)	<0.001
No	103 (37.7%)	152 (43.7%)	234 (56.5%)	

Notes: _χ_^2^ = Chi-squared test according to Pearson.

**Table 6:** Verbal abuse or violence experienced [number/month] by veterinarians at different workplaces.

Experiences of Verbal Abuse or Violence	Place of Work			p_KW_	p_B_
	Large City (*n* = 273)	Medium/Small Town (*n* = 348)	Rural Areal (*n* = 414)		
	Frequency (Number (%))				
Verbal abuse [number/moth]	3.2 ± 3.96 2 (0–22)	3.6 ± 4.12 2 (0–30)	3.2 ± 5.97 2 (0–50)	0.065	-
Violence [number/month]	0.1 ± 0.37 0 (0–3)	0.2 ± 0.78 0 (0–9)	0.1 ± 0.45 0 (0–4)	0.255	-

Notes: KW = Kruskal–Wallis test; B = Bonferroni correction.

## Data Availability

The datasets presented in this article are not readily available because the data contains potentially identifying information. Requests to access the datasets should be directed to correspondence author.

## Abbreviations

The following abbreviations are used in this manuscript:

AG	Age group
